# Senescence-Associated Alterations in Matrisome of Mesenchymal Stem Cells

**DOI:** 10.3390/ijms25105332

**Published:** 2024-05-14

**Authors:** Diana Matveeva, Daria Kashirina, Mariia Ezdakova, Irina Larina, Ludmila Buravkova, Andrey Ratushnyy

**Affiliations:** Institute of Biomedical Problems, Russian Academy of Sciences, Khoroshevskoye Shosse, 76a, 123007 Moscow, Russia; matveeva.dajana@yandex.ru (D.M.); daryakudryavtseva@mail.ru (D.K.); ezdakova.mi@gmail.com (M.E.); irina.larina@gmail.com (I.L.); buravkova@imbp.ru (L.B.)

**Keywords:** mesenchymal stem cells (MSCs), senescence, extracellular matrix (ECM), matrisome, senescence-associated secretory phenotype (SASP)

## Abstract

The process of aging is intimately linked to alterations at the tissue and cellular levels. Currently, the role of senescent cells in the tissue microenvironment is still being investigated. Despite common characteristics, different cell populations undergo distinctive morphofunctional changes during senescence. Mesenchymal stem cells (MSCs) play a pivotal role in maintaining tissue homeostasis. A multitude of studies have examined alterations in the cytokine profile that determine their regulatory function. The extracellular matrix (ECM) of MSCs is a less studied aspect of their biology. It has been shown to modulate the activity of neighboring cells. Therefore, investigating age-related changes in the MSC matrisome is crucial for understanding the mechanisms of tissue niche ageing. This study conducted a broad proteomic analysis of the matrisome of separated fractions of senescent MSCs, including the ECM, conditioned medium (CM), and cell lysate. This is the first time such an analysis has been conducted. It has been established that there is a shift in production towards regulatory molecules and a significant downregulation of the main structural and adhesion proteins of the ECM, particularly collagens, fibulins, and fibrilins. Additionally, a decrease in the levels of cathepsins, galectins, S100 proteins, and other proteins with cytoprotective, anti-inflammatory, and antifibrotic properties has been observed. However, the level of inflammatory proteins and regulators of profibrotic pathways increases. Additionally, there is an upregulation of proteins that can directly cause prosenescent effects on microenvironmental cells (SERPINE1, THBS1, and GDF15). These changes confirm that senescent MSCs can have a negative impact on other cells in the tissue niche, not only through cytokine signals but also through the remodeled ECM.

## 1. Introduction

Aging is defined as a decrease in functionality that occurs in all systems of the organism and leads to pathologies. Tissue homeostasis is maintained through the precise co-ordination of molecular signals in the cellular microenvironment. However, with age, the co-ordination deteriorates, and genetic and epigenetic changes accumulate in cells. There are several models of systemic aging. The stem cell theory of aging postulates that the organism dysfunction is attributable to the inability of various types of stem cells to sustain the replenishment of tissues with differentiated cells [1]. On the other hand, niche aging results in aberrant cell behavior [2].

Mesenchymal stromal/stem cells (MSCs) are adult stem cells with a unique affinity for damaged tissues and high proliferative and differentiation abilities. They have been shown to have reliable reparative properties, limiting apoptosis, enhancing angiogenesis, and demonstrating anti-inflammatory and immunoregulatory activity. These properties make MSCs a promising candidate for use in regenerative medicine and tissue engineering [3].

A significant number of researchers have attributed the beneficial effects of MSCs to their capacity to secrete biologically active factors, including cytokines and extracellular matrix (ECM) components. It is widely believed that these factors contribute to the therapeutic potential of MSCs. The ECM can influence the functional state of cells and modulate paracrine activity, as evidenced by studies [4,5,6]. The biological role of the ECM determines the integration of cells into tissues, tissues into organs, and organs in the organism. The ECM can directly influence cell activity through the interaction of structural glycoproteins with other ECM macromolecules, such as collagen, proteoglycans, and elastin, as well as the cytoskeleton, using transmembrane receptors like integrins. The molecular composition of the ECM affects cell migration and proliferation. The ECM plays a crucial role in maintaining the quiescent state of adult stem cells [7]. The ECM physically protects cells from differentiation signals by limiting the availability of growth factors and morphogens, while also facilitating the efficient nutrient exchange to support the long-term growth, survival, and multipotency of stem cells [8].

The term “matrisome” was proposed to describe the diverse and complex set of structural elements and associated molecules that constitute the ECM [9,10]. The matrisome is defined as the ensemble of over 1000 ECM proteins and ECM-associated proteins. The matrisome comprises core matrisome proteins, such as collagens, glycoproteins, and proteoglycans, as well as matrisome-associated molecules, including secreted factors, ECM regulators, and ECM-affiliated molecules. The investigation of the ECM from the perspective of matrisomal characteristics is currently a prominent area of research. This approach allows for a comprehensive description of the protein composition that determines the ECM’s function in different tissue niches or under specific experimental conditions.

Here, we must mention cell senescence. Cell senescence, characterized by an irreversible arrest of the cell cycle, occurs in response to endogenous and exogenous stresses, including telomere dysfunction, oncogene activation, and persistent DNA damage. At the same time, senescent cells remain active in terms of metabolism and demonstrate common phenotypic and molecular traits irrespective of their origin, including their flattened and enlarged morphology, increased cytoplasmic and lysosomal content, lipofuscin accumulation, resistance to apoptosis, disturbance of nuclear structure, formation of senescence-associated heterochromatin foci (SAHF), etc. [11]. Senescent cells via SASP contribute to impaired tissue regeneration, chronic age-related diseases, ageing, and even cancer progression [12,13]. On the other hand, senescent cells may accelerate wound healing and tissue repair under normal conditions [14]. Senescence is a powerful anti-cancer mechanism. It is activated in potential tumor cells and prevents dividing. Senescence is a cellular program that has both beneficial and detrimental effects on one’s health and lifespan. This phenomenon is considered an example of evolutionary antagonistic pleiotropy [15,16].

Senescence can be activated in response to variety stimuli, including changes in telomere structure, oxidative and genotoxic stress, oncogenic activation, epigenetic changes, chromatin disorganization, impaired proteostasis, mitogenic signals, nutrient deprivation, mitochondrial dysfunction, inflammation, signals of tissue damage, radiation, or chemotherapeutic agents. These different types of stress signals result in different types of senescence, such as telomere-dependent replicative aging, programmed aging, or non-telomeric stress-induced premature aging, including oncogene-induced aging (OIS), unresolved senescence caused by DNA damage, epigenetically induced senescence, and mitochondrial dysfunction associated with senescence. Recent studies have demonstrated that treatment with some chemotherapeutic drugs and ionizing radiations provoke “therapy-induced senescence (TIS)” in cancer cells. Genotoxic stimuli cause random damage to cellular macromolecules, leading to cell-to-cell variation in the aging phenotype despite a common program. Moreover, senescence is a complex and multistep process in which the properties of senescent cells continuously evolve and diversify depending on the microenvironment context [16,17].

The activation of the p53/p21WAF1/CIP1 and p16INK4A/pRB tumor suppressor pathways plays a central role in the regulation of senescence. The DNA damage response (DDR) is a signaling pathway in which ATM or ATR kinases block the cell cycle progression through the stabilization of p53 and the transcriptional activation of p21. However, senescence induced by E2F3 activation or c-Myc inhibition is DDR-independent and involves p19 and p16. After p16 or p53/p21 activation, cells progress to full senescence by downregulating lamin B1 (LMNB1), thereby triggering the extensive chromatin remodeling that underlies the production of an SASP, including pro-inflammatory cytokines, various growth factors, and proteases that, together, alter tissue structure and function. The progression to deep/late senescence may be driven by additional genetic and epigenetic alterations that result in further transcriptional changes and SASP heterogeneity. The SASP is one of the key features that distinguish senescent cells from quiescent, terminally differentiated, and other types of non-proliferating cells [15,16,18,19].

Similar to other cells, the activation of the senescent state of MSCs changes their morphological and functional characteristics. It is observed that there is an irreversible cell cycle arrest; an increased average cell size, granularity, and reactive oxygen species level, including anion superoxide; alterations in organelle morphology and activity; a specific gene expression; the appearance of heterochromatin foci (γH2AX); and a number of other hallmarks of cell senescence. It is believed that one of the important features of senescent MSCs is the decreased multipotency, which may restrict their reparative functions in tissues. Cell senescence contributes to dysfunctions in adipose-derived stromal/progenitor cell adipogenesis, triglyceride storage, and adipokine secretion. The question of changes in the osteogenic potential is still a controversial issue. SASP modifies the paracrine communication between MSCs and their physiological/pathological microenvironment. It should be noted that, depending on the tissue source and the method of senescence induction, the secretome composition may considerably vary. Moreover, MSCs obtained from different tissues may respond to stress inducers with different levels of sensitivity. Some authors demonstrated that p16INK4A is a key factor in the regulation of human MSC growth, and, most importantly, the careful monitoring of DNA methylation should be considered during the culture of hMSCs [20,21,22,23,24].

The activation of cellular senescence is associated with significant alterations in the secretion profile of cells. Consequently, the composition of the ECM and its properties will also undergo changes. The direct impact of senescent cells on tissue formation, beyond paracrine signaling, remains unclear, as noted by other authors [25]. It is evident that various cell types respond differently to senescence. This work primarily focuses on MSCs, which play a crucial role in regulating tissues and maintaining homeostasis in numerous tissue niches. Therefore, the analysis of the ECM of senescent cells can offer valuable insights into the pathophysiological regulation of MSCs and aid in optimizing therapeutic protocols. In a broader sense, understanding the processes occurring in aging cells and identifying potential targets to slow down or stop the aging process is crucial. The objective of this study was to investigate the aging-associated changes in the matrisome of MSCs.

It is suggested that the aging of the ECM may be more significant than the aging of the cells themselves. This is because there are more effective mechanisms for the restoration and removal of damaged proteins and organelles inside the cell, in contrast to the ECM. The age-related dysfunction of the ECM is often associated with age-related abnormalities and pathologies, such as fibrosis, cardiovascular disease, and neurodegenerative diseases. Further research in this direction is necessary [26].

## 2. Results

As part of the study, cultures of senescent MSCs were obtained, the morphological characteristics of the ECM and the expression of individual genes were studied, and a proteomic analysis of the matrisome was performed. Matrisome proteins were separately detected in the ECM, cell lysates, and conditioned medium.

### 2.1. Cell Senescence Induction

The DNA alkylating agent mitomycin C (MmC) was used to produce senescent MSCs. To date, it has been demonstrated in primary and immortalized cultures (human dermal fibroblasts; alveolar epithelial cells; non-small-cell lung cancer—A549 cells; and primary and telomerase immortalized human corneal limbal epithelial cells) that MmC-treated cells display characteristics of senescent cells, such as abrogated proliferation, a stable change in cell morphology, intensified senescence-associated β-galactosidase (SA-β-gal) activity, and a transient increase in the ROS level, upregulated SASP factors, p21, p53, retinoblastoma (Rb) genes, etc. [27,28,29,30]. MmC is an alkylating DNA-damaging agent and ROS producer. ROS alone is not sufficient to induce a permanent senescence, while DNA damage by alkylating agents is needed to induce the permanent senescent cell type. Alili et al. note that this drug-induced accelerated senescence is comparable with replicative senescence [28]. Gorgoulis et al. include MmC in the “Selected List of Factors Triggering Senescence” as a DNA cross-linker [15]. Alkylating agents have been used extensively for the anti-cancer treatment. These agents can induce genotoxic stress by initiating a nucleophilic attack against guanine bases by attaching an alkyl group to nitrogen atom number 7, thereby resulting in DNA cross-linking [31]. hTERT-immortalized fibroblasts and cancer cells respond to genotoxic agents like elevated ROS, ionizing radiation, or MmC by upregulating genes associated with senescence. The expression of human telomerase (hTERT) does not prevent stress-induced senescence [29,32].

After MmC treatment, the cells were cultivated without passing 10 days. This period is necessary for the full implementation of the program of senescence. Similar exposures are used in other studies using stress-induced senescence [33]. On the other hand, a long-term expansion without passing is necessary to form an ECM layer under cells.

**Figure 1:**
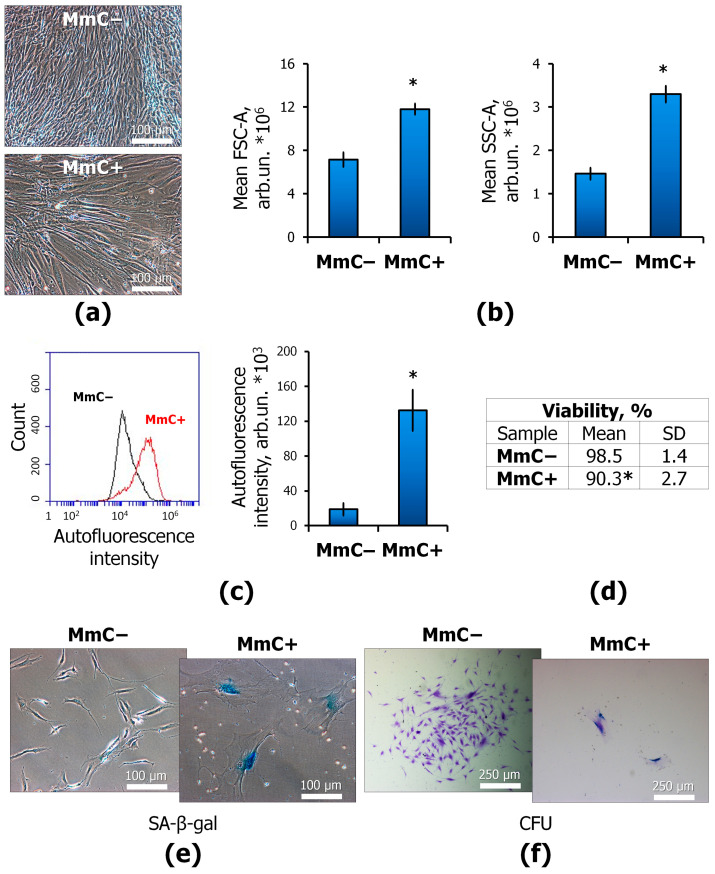
Senescence markers of MmC-treated MSCs: (**a**) cell monolayer morphology, and light microscopy; (**b**) forward scattering (FSCA) and side scattering (SSC-A) to illustrate size and cytoplasm vacuolization of MSCs, respectively, and flow cytometry; (**c**) autofluorescence intensity, and flow cytometry; (**d**) cell viability, annexin/propidium iodide (Ann/PI) staining, and flow cytometry; (**e**) histochemical evaluation of senescence-associated-β-galactosidase (SA-β-gal) activity in MSCs; and (**f**) colony-forming unit assay, and light microscopy. Data are shown as mean ± SD; *n* ≥ 4, * *p* < 0.05. MmC—mitomycin C, MSCs—mesenchymal stem cells.

Ten days after exposure to MmC, MSCs significantly changed its morphology (Figure 1a,b). An increase in the volume and granularity of the cytoplasm was noted. An evaluation of the morphological indicators FSC-A and SSC-A revealed an increase in these indicators by 40% and 140%, respectively. Treatment with MmC led to an increase in cell autofluorescence (Figure 1c). This may indicate lipofuscin accumulation. Autofluorescence is a reliable in vitro marker of cell senescence in MSCs [34]. At the same time, the vitality of cells after exposure decreased by approximately 10% (Figure 1d). It is likely that the reduced vitality is due to the increased vulnerability of the cells during sample preparation for flow cytometry analysis. A significant increase in cell volume increases the risk of damage during manipulation. A Petri dish (60 mm) was seeded with 250,000 cells. An increased cell number (2.9 ± 0.7 fold) was observed in the MmC− MSCs after 10 days. The number of cells in MmC+ samples did not change over 10 days.

To confirm the senescent state, the activity of SA-β-gal was assessed. This approach to confirming cellular aging is one of the most widely used. The SA-β-gal is a lysosomal hydrolase activated at pH 4. Nevertheless, it was shown that SA-β-gal may be activated at pH 6 in senescent cells [35]. Both MmC− and MmC+ cells were passed to new culture dishes for 48 h to avoid the effect of a dense monolayer on the analysis results. In the MmC+ group, 100% of cells were active in this enzyme, while no (0%) SA-β-gal-positive cells were detected in the intact MSCs (Figure 1e).

The main sign of cellular senescence is the permanent arrest of the cell cycle. To assess the cell proliferative activity, the MSCs were seeded at a low density on new culture dishes (100 cells per 60 mm dish). After 7 days, colony formation was assessed (Figure 1f). It has been shown that treated cells are not able to form new colonies. This observation confirms cell cycle arrest.

Taken together, these data indicate the activation of the senescent state of MSCs after MmC treatment under experimental conditions.

### 2.2. Morphological Characteristics of “Senescent” ECM and Gene Expression Analisis

**Figure 2:**
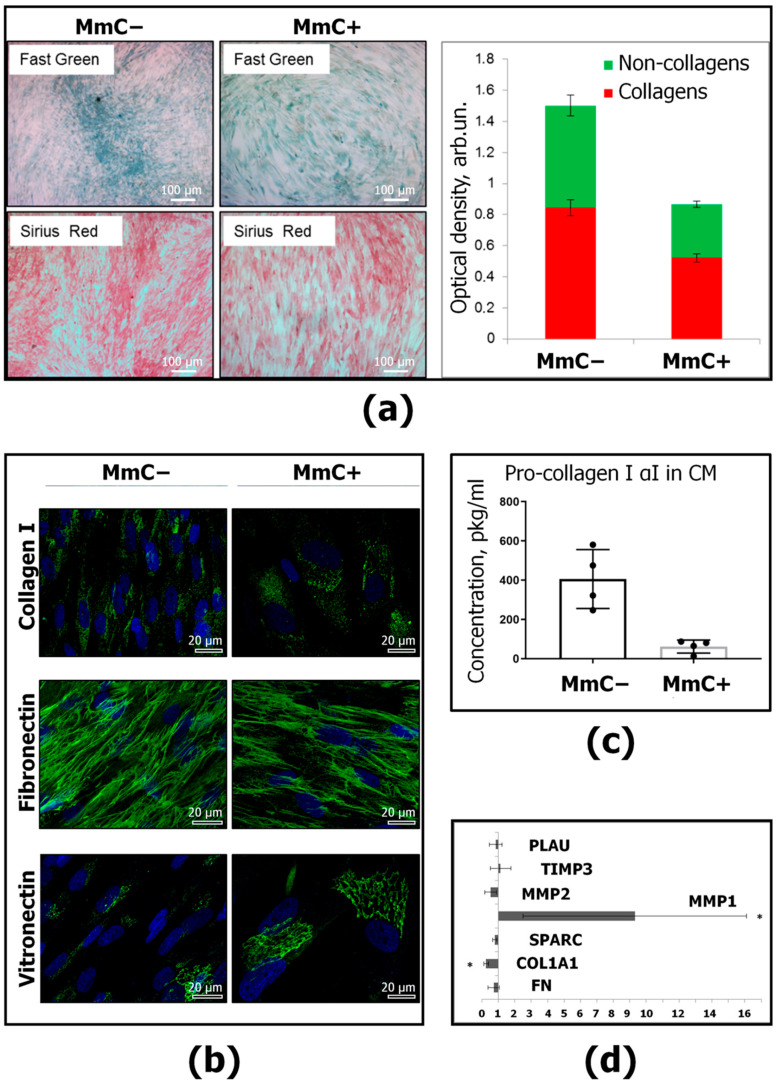
Characteristics of ECM proteins produced by young (MmC−) and senescent (MmC+) MSCs: (**a**) histological identification of collagenous (Sirius Red) and non-collagenous (Fast Green) proteins and their semiquantitative spectrophotometric determination, bar—50 µm; (**b**) immunocytochemical detection of ECM proteins (ECM proteins—green, DAPI—blue), representative microphotographs, bar—20 µm; (**c**) the content of pro-collagen 1α1, detected by enzyme immunoassay, in the conditioned medium of young and senescent MSCs; data are presented as M ± SD, *p* ≤ 0.05, *n* = 3; (**d**) relative gene expression (2^−ΔΔCt^) in MmC+ versus MmC− MSCs encoding ECM proteins: type I collagen (*COL1A1*), fibronectin (*FN1*), osteonectin (*SPARC*), metalloproteinases (*MMP1*, *MMP2*), tissue inhibitor of metalloproteinases (*TIMP3*), and urokinase plasminogen activator (*PLAU*); data normalized to the expression of *HPRT1*, *RPLP0*; * *p* ≤ 0.05, *n* ≥ 3.

In order to characterize the total production of ECM proteins, histological dyes were used. Sirius Red F3BA and Fast Green FCF dyes specifically bind to collagenous and non-collagenous proteins, respectively (Figure 2a). For semiquantitative analysis, the stains of the culture plates were eluted and the absorbance was determined using a PR 2100 plate photometer (Bio-Rad Laboratories, Berkeley, CA, USA). In senescent MSCs, the total production of ECM proteins was reduced, with an accumulation of collagenous proteins 1.6 times less and non-collagenous proteins 1.9 times less compared to intact MSCs.

Furthermore, the presence of collagen type I molecules and adhesive glycoproteins, such as fibronectin and vitronectin, was identified (Figure 2b). Collagen I is a major fibrillar collagen, widely distributed in various tissues as the main structural protein. Fibronectin is an ECM glycoprotein that mediates interactions with integrins. Vitronectin is a key regulator of mammalian tissue repair and remodeling activity. It is noteworthy that both MmC− and MmC+ MSC exhibit the synthetic activity of the investigated molecules. In senescent cultures, collagen I was heterogeneously distributed between cells and was detected in smaller quantities. Fibronectin molecules formed a uniform dense network in both young and senescent cultures. Vitronectin was localized predominantly in the endoplasmic reticulum, and its expression was more significant in senescent MSCs.

To quantify the level of collagen production, we examined the secretion of pro-collagen 1α1 in the conditioned medium using an enzyme-linked immunosorbent assay (ELISA) (R&D Systems, Minneapolis, MN, USA) and the expression of the *COL1A1* gene. The content of pro-collagen 1α1 in the conditioned medium from senescent MSCs was significantly reduced (10 times) compared to “young” MSCs (Figure 2c). Additionally, a significant decrease in the transcription of *COL1A1* was revealed (Figure 2d).

A study of other genes encoding ECM structural molecules showed that the expression of fibronectin and osteonectin did not change during senescence. Among the regulatory molecules, a significant increase in the expression of collagenase *MMP1* was noted in MmC+ compared to the MmC− group of MSCs. No significant differences were found in the transcription of *MMP2*, *PLAU,* and *TIMP3* (Figure 2d).

A study of other genes encoding ECM structural molecules demonstrated that the expression of fibronectin and osteonectin remained unaltered during senescence. Among the regulatory molecules, a notable increase in the expression of collagenase *MMP1* was observed in MmC+ compared to MmC− MSCs. No significant differences were identified in the transcription of *MMP2*, *PLAU*, and *TIMP3* (Figure 2d).

### 2.3. Senescence-Related Changes in Proteome Composition of MSC Matrisome

At the subsequent stage of investigation, it was necessary to identify differences in the matrisome of senescent MSCs. Using liquid chromatography–mass spectrometric analysis, 192 proteins related to the human matrisome were identified in samples of the decellularized extracellular matrix (dcECM), cell lysate, and MSC secretome (MatrisomeBD) [36,37]. In each group, the differences between the MmC− and MmC+ subgroups were analyzed separately. Semi-quantitative protein analysis was performed using PEAKS Studio 8.5 software, which provided data on the relative content of proteins in the samples. Using statistical tests, we identified proteins with altered levels in senescent MSCs (MmC+) compared to intact ones (MmC−). Subsequently, we characterized the proteins for each group of matrisomes.

**Figure 3:**
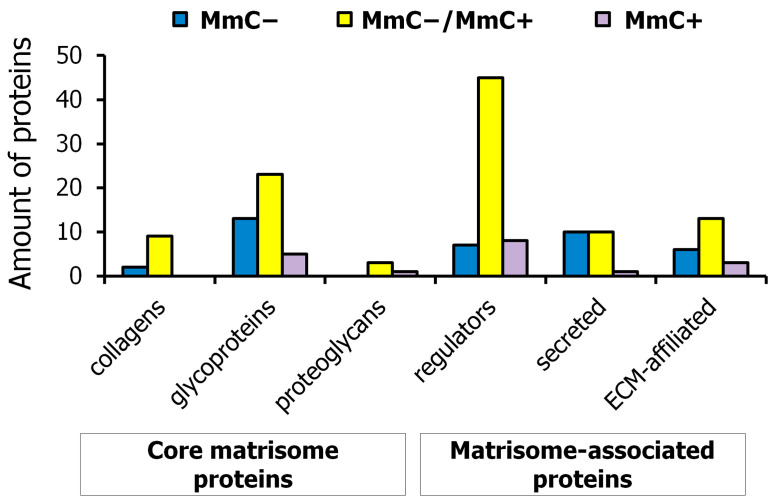
Quantitative distribution of MSC proteins identified by mass spectrometry in samples of young (MmC−), senescent (MmC+), and both cell types (MmC−/MmC+) by functional groups of the matrisome.

In addition to examining the relative protein content of the samples, we also investigated the presence of proteins. Upon the distribution of proteins into groups, it was observed that unique proteins within the MmC− MSC matrisome were predominantly identified among glycoproteins and secreted factors, while, in the MmC + MSC matrisome, they were primarily found among proteoglycans and regulatory molecules (Figure 3); 34% of the proteins that were identified in the comparison groups belong to the core matrisome, and 66% to matrisome-associated molecules. The largest numbers of altered proteins in the senescent dcECM were glycoproteins and regulatory molecules.

**Figure 4:**
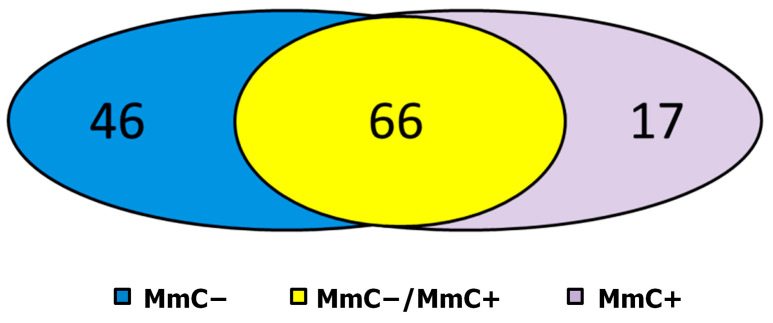
Venn diagram analysis. Graph show common and specific proteins among dcECM obtained from intact (MmC−) and senescent (MmC+) MSCs analyzed by mass spectrometry.

In MmC− dcECM samples, 112 proteins were identified. In MmC+ dcECM samples, 82 proteins were identified. Of these proteins, 66 were detected in both experimental groups (Figure 4).

**Figure 5:**
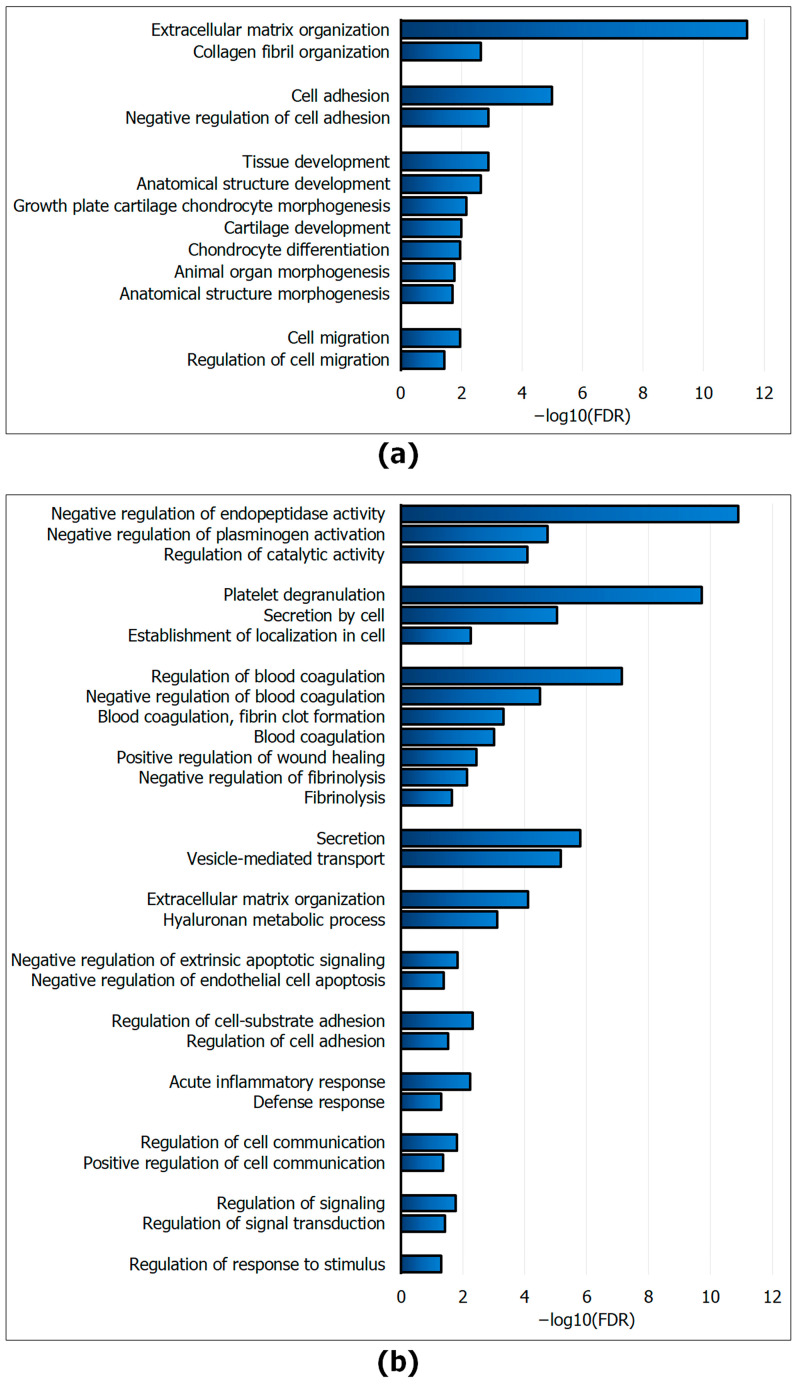
Gene Ontology functional enrichment analysis of the biological processes: (**a**) unique and upregulated matrisome proteins in MmC− dcECM samples; (**b**) unique and upregulated matrisome proteins in MmC+ dcECM samples. dcECM—decellularized extracellular matrix.

After analyzing the functional annotation of proteins in the GO and WikiPathways databases using the String web resource (https://string-db.org/; accessed on 12 February 2024), we formed two groups of molecules (Figure 5). Group 1 included specific and upregulated proteins in MmC− dcECM samples. Group 2 included specific and upregulated proteins in MmC+ dcECM samples. In the “young” ECM, proteins responsible for the organization of the ECM (GO:0030198), in particular, collagen fibers (GO:0030199), and molecules involved in adhesion (GO:0007155), cell migration (GO:0016477), and their regulation (GO:0007162 and GO:0030334, respectively) were identified. A considerable number of matrisome proteins were identified as being involved in the morphogenesis of anatomical structures (GO:0048856) and organs (GO:0009887), and tissue development (GO:0009888), with a particular focus on cartilage. Additionally, the presence of tissue (GO:0003429, GO:0051216, and GO:0060351), including chondrocyte differentiation (GO:0002062), was observed (Figure 5a).

A significant number of molecules present in the senescent ECM are involved in the modulation of cellular behavior (Figure 5b). For instance, a cluster of molecules involved in the regulation of platelet degranulation (GO:0002576), blood coagulation (GO:0072378, GO:0007596, and GO:0007596), fibrinolysis (GO:0042730, and GO:0051918), and wound healing (GO:0042730, and GO:0051918) were annotated. The following annotations were made: GO:0030193, GO:0030195, GO:0042730, GO:0051918, and GO. Molecules regulating catabolic activity (GO:0050790) have been identified, in particular, those that inhibit endopeptidase catalytic activity (GO:0010951) and plasminogen activation (GO:0010757). Additionally, this group of proteins plays a role in the metabolism of hyaluronan (GO:0030212) and inflammatory responses (GO:0002526, and GO:0002920). A number of proteins were identified that are involved in regulating cell communication (GO:0010646, and GO:0010647), adhesion (GO:0010810, and GO:0030155), and signaling (GO:002305). The following GO terms were revealed: GO:0009966, GO:0023056, GO:0051897, GO:1902041, and GO:2001237.

**Figure 6:**
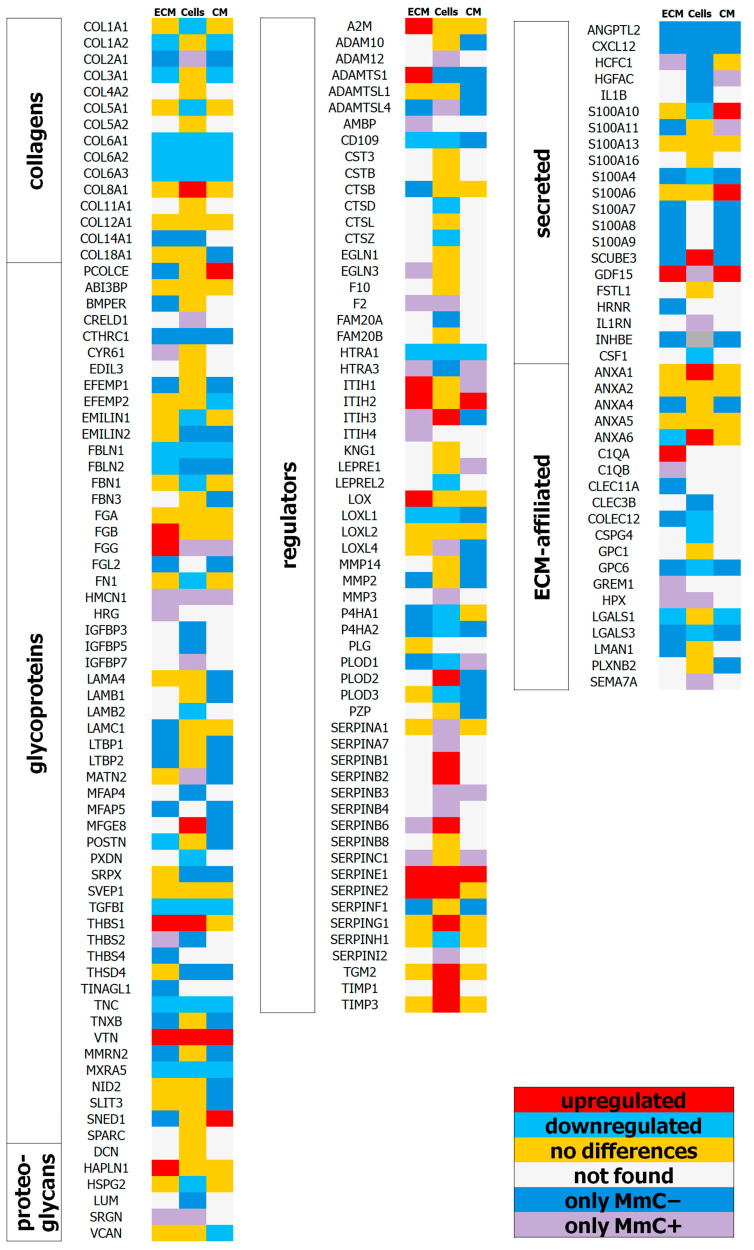
Matrisome proteins identified in dcECM, cell lysate, or conditioned medium of MmC− and MmC+ MSCs. The heat map reflects the direction of change in the relative content of proteins in MmC+ compared to MmC− samples.

Subsequently, we will examine the identified alterations in the matrisome, not only in the ECM, but also in the cell lysates and in the conditioned medium (Figure 6).

#### 2.3.1. Core Matrisome Protein

Collagens: A significant decrease in the levels of fibrillar (COL1A2, and COL3A1) and network-forming (COL6A1, COL6A2, and COL6A3) collagens was observed in both the dcECM and the conditioned medium of senescent MSCs. The levels of fibrillar collagens COL1A1 and COL5A1 were reduced only in the cell lysates of MmC+ MSCs. Collagens that are unique to young MSCs and accumulate in the ECM include cartilage-tissue-specific COL2A1 and fibril-associated COL14A1.

Glycoproteins: After being treated with MmC, glycoproteins that are typically involved in cell adhesion (LAMC1, LAMB1, LAMA4, NID2, TNXB, THBS4, EMELIN2, and SRPX) and calcium ion binding (FBN3, and EFEMP1) are no longer detectable in probes. Furthermore, growth factors (LTBP1, LTBP2, IGFBP3, and IGFBP5) and heparin (PCOLCE, LTBP2, THBS4, TNXB, and SLIT3) are no longer detectable in the probes. Glycoproteins that are specific to MmC+ samples are involved in blood coagulation (FGG, and HRG), growth factor binding (CYR61, and IGFBP7), and chemotaxis (CYR61, HRG).

Downregulated glycoproteins in MmC+ samples belong to structural and adhesion molecules (FBLN1, POSTN, TNC, and LAMB2). A separate cluster of molecules is associated with the formation of elastin fibers (FBN1, FBLN2, EMELIN1, and EFEMP2). Interesting that molecules with a reduced level of expression in senescent MSCs were PXDN, which is involved in the metabolism of peroxide compounds in the cardiovascular system [38], as well as MXRA5, which has anti-inflammatory and antifibrotic properties, limiting the induction of the expression of chemokines, fibronectin, and collagen in response to TGF-β and inflammatory stimuli.

The upregulated glycoproteins in the samples of senescent cells were structural and adhesive vitronectin (VTN) and the matricellular protein thrombospondin-1 (THBS1 or TSP-1). Increased levels of lactadherin (MFGE8) have been observed, which promotes the phagocytic clearance of apoptotic cells in many tissues [39].

Proteoglycans: An increase in the content of linker proteoglycan that binds hyaluronic acid (HAPLN1) was observed in the dcECM of senescent MSCs. This proteoglycan colocalizes with collagen molecules and provides resistance to compression in tissues. A specific proteoglycan, serglycin (SRGN), was identified as playing an important role in the formation of secretory granules and mediating MMP2 processing. Among the proteoglycans whose levels were reduced in MmC+ MSCs, a significant decrease was observed in perlecan (HSPG2) in the cell lysate and versican (VCAN) in the conditioned medium. Perlecan is a key adhesion protein of the basement membrane that is involved in the regulation of vasculogenesis [40]. Previously, our work showed that the senescent secretome has reduced angiogenic properties [41].

#### 2.3.2. Matrisome-Associated Proteins

ECM REGULATORS: The cell lysates of senescent MSCs exhibited a reduction in the content of enzymes responsible for the organization of collagen fibrils, including PLOD1, PLOD3, P4HA1, P4HA2, LOXL1, and LEPREL2. Additionally, a decrease in lysates was observed for cathepsins (CTSD and CTSZ) and SERPINH1, a chaperone in the collagen biosynthesis pathway [42].

In probes from senescent MSCs, the decreased accumulation of the serine protease HTRA1 and serine-type endopeptidase inhibitor (CD109) was observed. Both enzymes are involved in the inhibition of TGFβ signaling. A recent study demonstrated a link between β3 integrin and senescence through the activation of the transforming growth factor–β pathway [43].

ADAMTSL4 involved in the biogenesis of fibrillin microfibrils (FBN1), the angiogenesis inhibitor SERPINF1, and the matrix metalloproteinases MMP2 and MMP14 were detected only in the dcECM of intact MSCs.

In the dcECM of senescent cells, a significant increase in proteins involved in fibrolysis reactions (A2M, SERPINE1, and SERPINE2); inter-alpha-trypsin inhibitors heavy chain (ITIH1, and ITIH2), which bind hyaluronan and are involved in the metabolism of hyaluronic acid; and metalloproteinase ADAMTS1, which has angiogenesis inhibitory activity and collagen cross-linking lysyl oxidase (LOX) was observed. Furthermore, the presence of hyaluronan-binding proteins (IH2), metalloproteinase ADAMTS1 with angiogenesis inhibitory activity, and collagen cross-linking lysyl oxidase (LOX) was identified. The levels of ITIH3, serine protease inhibitors (SERPINB1, SERPINB2, SERPINB6, and SERPING1), metalloproteinase inhibitors (TIMP1, and TIMP3), and transglutaminase (TGM2) were found to be elevated in the cell lysates of senescent cells.

SECRETED FACTORS: The detection of growth factors and cytokines is limited by several factors, particularly the sensitivity of the method [44]. A number of unique or elevated proteins have been identified in intact MSCs. The predominant accumulation of calcium-binding proteins (S100A), ANGPTL2, vascular-sprouting-inducing protein, and chemokine CXCL12 was observed. Concurrently, a notable accumulation of the growth factor GDF15 was observed within the senescent ECM.

ECM-AFFILIATED PROTEINS: There was a significant decrease or absence of ECM-affiliated proteins in senescent MSCs. These included membrane-bound proteoglycans GSPG4 and GPC6, and lectins LGLAS1, LGALS3, and COLEC12. Functionally, most of them (GSPG4, GPC6, and LGALS3) are involved in cell migration.

Unique to senescent dcECM, GREM1 was identified. GREM1 can inhibit the growth and osteodifferentiation of normal cells, as well as reduce viability [45].

## 3. Discussion

In our study, we investigated the matrisome of MSCs in stress-induced senescence caused by the mitomycin (MmC) treatment of cells. At the initial stage of the work, it was necessary to obtain senescent MSCs. MmC generates oxygen radicals (ROS) and alkylates DNA, thereby promoting inter- and intrachain cross-links. Ten days after MmC treatment, characteristic morpho-functional changes were revealed. The volume and granularity of the cytoplasm increased, as did the activity of SA-β-gal. The cells do not form a colony.

Furthermore, the matrisome of senescent MSCs was investigated. A proteomic analysis revealed a decrease in the level of fibrillar and adhesive collagens in conditioned the medium, in the dcECM, and in the cell lysates. Additionally, a decrease in the number of enzymes involved in the post-translational modification of collagens was observed in the cell lysates (PLOD1, PLOD3, P4HA1, P4HA3, LOXL1, and LEPREL2). The findings indicate a decrease in collagen production. Histologic staining confirmed a significant decrease in the total production of both collagenous and non-collagenous proteins. A more detailed analysis of key collagen I type revealed a decrease in pro-collagen 1α1 levels in the conditioned medium using ELISA. A real-time PCR showed a decrease in the transcription of COL1A1. Concurrently, an increase in the expression of the interstitial metalloproteinase gene (MMP1) with a substrate specificity for type 1 collagen was established. MMP1 is one of the components of SASP and is considered one of the significant biomarkers of aging [46].

Other studies have indicated that changes occur in the ECM of senescent fibroblasts in some models of aging. These changes include the increased expression of proteolytic enzymes (matrix metalloproteinases, adamalysins, urokinases, and cathepsins) and the decreased production of structural components of the ECM (collagens, glycoproteins, and proteoglycans) [47,48]. Such changes may underlie both physiological and pathological processes. In the first case, the senescent state is one of the mechanisms of cell elimination, for example, to regulate the number of myofibroblasts, the precursors of which can be MSCs. The elimination of myofibroblasts, a decrease in ECM deposition, and an increase in its degradation are necessary at the final stages of wound healing to avoid the development of fibrotic conditions [49]. On the other hand, the accumulation of senescent cells in tissues with the ineffective production of the structural components of ECM or its excessive degradation may lead to tissue pathologies. The participation of MSCs in senescence-associated ECM remodeling is consistent with the context of connective tissue aging, including atherosclerosis, osteopenia and osteoporosis, osteoarthritis, tendon dysfunction, and age-related disorders of the spine and joints [50].

The alterations in the composition of the crustal matrisome observed in senescent MSCs may be responsible for the age-related decline in tissue elasticity and the thinning of the basal membrane [51]. A reduction in the levels of major fibrillar collagens of healthy bones, tendons, and ligaments (type I and III collagens), glycoproteins associated with elastin fibres (FBN1, FBLN2, EMELIN1, and EFEMP2), and ADAMTSL4, which promotes the biogenesis of fibrillin microfibrils, was observed. Additionally, a decline in the levels of several structural proteins of basal membranes (FBLN1, COL6A1, and TNC) was found.

The GO and WikiPathways databases indicate that core matrisome proteins, which exhibit decreased levels upon exposure to mitomycin C, are involved in the formation of focal contacts, transmission of the PI3K/AKT/mTOR signaling pathway, and binding of platelet-derived growth factor and other growth factors. This suggests that these functions are suppressed during senescence. These molecules play an important role in adhesion, as well as proliferation and growth [52,53,54]. The mechanisms by which ECM proteins can affect this diversity of functions are mediated by interactions with a variety of cellular receptors, including integrins. Dynamic receptor–ECM interactions promote cycles of cell adhesion and cell migration. Depending on the biomechanical properties of the ECM, after anchoring, various signaling pathways involved in intracellular signaling and mechanotransduction are triggered in cells, which controls cell behavior by changing the gene expression.

Experiments on the recellularization of senescent cells on the “young” ECM demonstrated a slowing of the signs of senescence. The work of Choi et al. showed that senescent fibroblasts, which were seeded on the ECM from fibroblasts at early passages, had a reduced expression of β-galactosidase, a decrease in reactive oxygen species, a restoration of mitochondrial potential, and an elongation of telomeres [55]. A reduction in the level of reactive oxygen species by 30–50% was observed in MSCs derived from the bone marrow of mice when cultured on ECM from young individuals (3 weeks) in comparison to ECM from old individuals (18 weeks) [56]. The cultivation of senescent MSCs from synovial fluid on fetal dcECM has been demonstrated to enhance their ability to differentiate in chondrogenic and adipogenic directions [57]. The observed effects can be attributed, at least in part, to the interaction of various adhesion molecules that are essential for cellular functioning. This interaction is diminished in the senescent ECM.

In our study, a reduced number of molecules with cytoprotective and anti-inflammatory properties (Collagen VI, fibulin 1, MXRA5, etc.) was found in extracts of senescent ECM compared to the ECM of “young” MSCs. Collagen VI has a wide range of effects on cells, including the counteraction to apoptosis and oxidative damage, the regulation of autophagy and cell differentiation, and the maintenance of satellite cell stemness in muscle tissue [58,59]. It is hypothesized that MXRA5 may possess anti-inflammatory and anti-fibrotic properties, as evidenced by its ability to limit the induction of chemokines, fibronectin, and collagen expression in response to TGF-β1 and pro-inflammatory stimuli [60]. It is conceivable that these molecules, which are characteristic of the young ECM, may influence the cells that are seeded on it in a manner that is beneficial to the cells and has an opposite effect to that of aging.

The identification of ECM-dependent signaling mechanisms triggered by the recellularization to the senescent ECM remains a relevant topic for the study of the mechanisms of niche aging. A number of studies have demonstrated the opposite effect, with a decrease in cell proliferation during the recellularization of the senescent ECM [61,62]. This phenomenon may be attributed to alterations in the ECM rigidity, the accumulation of regulatory molecules within the ECM, and the deposition of cytokines—SASP components—within the ECM.

In our research, we employed a mass spectrometric analysis to identify a number of molecules that exhibited upregulation in senescent ECM. The increased accumulation of these molecules can potentially lead to the disruption of tissue homeostasis or contribute to the senescence of other cells in the niche. Among the proteins identified in this experiment are Cyr61, HAPLN1, and ADAMTS-1. The matricellular proteins Cyr61 and CTGF have been demonstrated to induce cellular senescence in fibroblasts by binding to β1 [63,64]. The expression of HAPLN1 has been observed to be increased in several types of musculoskeletal diseases, including rheumatoid arthritis. Chen et al. (2022) first demonstrated the function of HAPLN1 in stimulating the proliferation and inflammatory phenotype of fibroblast-like synoviocytes [65]. ADAMTS-1 is a unique protein of the metalloproteinase family that is anchored in the ECM [66]. ADAMTS-1 significantly blocks VEGFR2 phosphorylation by directly binding and sequestering VEGF-165, thereby suppressing endothelial cell proliferation [67].

It is noteworthy that, in the senescent ECM, there is an increased accumulation of proteins belonging to the fibrinolytic system (A2M, PAI-1, and Vitronectin). These proteins are physiologically designed to eliminate the fibrin clot, converting plasminogen into plasmin. Nevertheless, contemporary research has demonstrated the involvement of the fibrinolytic system in the recruitment of inflammatory cells/MSCs and the regulation of proteolytic activity in wound healing and the regeneration of mesodermal tissues (bone, cartilage, and muscle) [68].

Plasminogen activation through the interaction of urokinase-type plasminogen activator with its receptor (uPA-uPAR) facilitates cell migration by increasing pericellular proteolysis [69] uPA-uPAR binding promotes cell adhesion. uPAR lacks transmembrane and intracellular domains, so it requires coreceptors such as integrins or vitronectin to trigger signaling pathways that are involved in cell proliferation/survival (RAS-ERK 1/2, and Akt-PI3 K), differentiation (RhoA-ROCK), and apoptosis (FAK-JNK/p38). PAI-1 has an anti-adhesive effect because it has a higher affinity for vitronectin and can compete for uPAR binding to vitronectin [70]. Impaired wound healing in diabetic patients has been associated with elevated levels of circulating PAI-1 [71]. In addition, there is evidence that PAI-1 is the major promoter of vascular pathologies, including arterial thrombosis and perivascular fibrosis [72,73]. Thus, transgenic mice with an overexpression of PAI-1 develop atherosclerosis with age, while animals with a PAI-1 deficiency are protected from experimentally induced vascular diseases [72]. Perhaps suppressing the expression or function of PAI-1 could be one of the approaches to the treatment and prevention of age-associated diseases, including thrombosis, fibrosis, diabetes, and others [74].

MSCs are one of the cellular components of the hematopoietic niche in the bone marrow [75]. MSCs are also found in various tissues, where they interact with perivascular cells and can adequately respond to signals during tissue damage. The interaction of cellular and non-cellular components in a niche forms a regulatory network due to contacts, the ECM–cell interaction, and paracrine and autocrine factors [7,76]. In our study, CXCL12 was not detected either in the conditioned medium or in the ECM extract in the group of cells exposed to MmC, although, in the young MSCs, CXCL12 was detected in all types of samples, indicating a significant suppression of its secretion in senescent MSCs. In the bone marrow niche, CXCL12 plays an important role in maintaining the quiescence, self-renewal, and retention of HSCs in the niche. CXCL12 is a potent chemoattractant for endothelial cells. Its products ensure the involvement of MSCs in reparative processes, including wound healing. Such changes may characterize one of the possible mechanisms of niche aging, leading to the disruption of homeostasis and the development of age-related tissue pathologies.

A number of molecules have also been identified that are considered as markers of senescence, including SERPINE1 (Plasminogen activator inhibitor 1, PAI-1), SERPINE2, THBS1, and GDF15 [77]. The increase in PAI-1 expression in our experiment is predictable, since the gene of this protein is a target for the p53 pathway, which is activated as a result of DNA damage by MmC. It is known that PAI-1 is a component of the senescence-associated secretory phenotype (SASP) [78]. It is important to note that PAI-1 is not just a biomarker of the aging phenotype, but is necessary and sufficient for the induction of replicative senescence upon the activation of p53 [79]. At the same time, PAI-1 can independently cause cellular senescence [80,81], being a key inducer of the aging program [82]. In addition, PAI-1 is consistently among the genes with the highest activation in all models of induced senescence [22]. Based on these results, it can be assumed that PAI-1, secreted by senescent cells, can indeed serve as a mediator of paracrine aging. It is suggested that the TGF-β1 pathway plays an important role in the induction of aging through PAI-1. PAI-1 is a known target gene of the TGFβ1 pathway [83]. In addition, PAI-1 itself stimulates the synthesis of TGF-β1 [84].

Data have been obtained indicating that TGF-β1 induces cell aging by stimulating reactive oxygen species and the expression of SASP factors, including PAI-1 [85,86]. Thus, we can hypothesize that PAI-1 is a key regulator of aging, both autocrine and paracrine, and the increased expression of PAI-1 will contribute to a continued increase in the production of both PAI-1 and other regulators of aging, including TGF-β1.

The involvement of the TGF-β1 signaling pathway in the aging process is indicated by another extracellular matrix protein, the concentration of which was increased in our study—thrombospondin-1. THBS1 modulates MSC proliferation through the TGF-β pathway [87]. THBS1 levels are known to increase in response to injury and during development. THBS1 activates latent TGF-β1 by releasing mature TGF-β1 [83]. In human endothelial cells, THBS1 promotes aging and arrests the cell cycle. At the molecular level, THBS1 increases the generation of reactive oxygen species (ROS), which leads to an in-crease in the amount of the p53 transcription factor. p53 mediates the DNA damage response that leads to senescence through the activation of Rb and p21 (CDKN1A gene), which inhibit the cell cycle progression. Mice lacking THBS1 showed a decreased ROS production, p21 expression, p53 activity, and signs of aging. Conversely, an increase in the amount of THBS1, p53, and p21 was observed in the lung tissue of aging people. Thus, THBS1 may be a potential target for controlling the aging process at the molecular level [88].

The protein GDF15, which also regulates the TGF-β1 signaling pathway, transmits the SMAD proteins’ signal, and negatively regulates the growth hormone receptor signaling pathway, deserves special attention. Many cell types produce GDF15 in response to oxidative stress or inflammatory signaling molecules. It was shown that GDF15 levels increased during cellular senescence induced by ionizing radiation, and the inhibition of GDF15 partially prevented radiation-induced cellular senescence. Conversely, GDF15 promoted the induction of cellular senescence in HAECs, which was confirmed by the G0/G1 cell cycle arrest, decreased cell proliferation, and increased senescence-associated β-gal staining, and was accompanied by the development of oxidative stress [89]. GDF15 is currently considered as a biomarker of cardiovascular diseases [90] and even as a predictor of disease progression and mortality [91]. Perhaps its involvement in human diseases is caused by its ability to induce aging processes.

The above examples of ECM protein accumulation contributing to fibrosis correlate with the previously described phenomenon of fibrogenesis accompanying the development of fibrosis in age-related tissues: lung, heart, kidney, and liver [92,93,94,95]. Mass spectrometric analysis showed that the stress-induced senescence of MSCs leads to the increased production of ECM regulatory enzymes (TGM2, and LOX), responsible for the formation of intra- and intermolecular covalent bonds (crosslinks) between ECM components, such as fibrillar collagens and elastin [96,97]. The development of tissue fibrosis leads to the excessive accumulation and disruption of the mechanical homeostasis of the ECM, which entails the mechano-activation of the pro-fibrotic signaling pathways [98].

**Figure 7:**
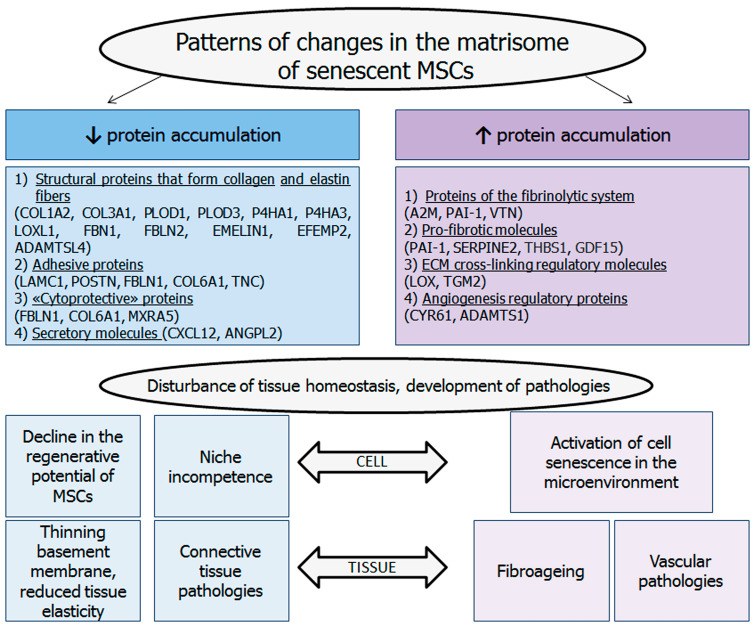
Matrisome analysis of senescent MSCs revealed the main patterns of changes in the produced ECM. A shift in production towards regulatory molecules and significant downregulation of the main structural and adhesion molecules of the ECM have been established. Such changes can contribute to the deterioration of cell function, the activation of sensory phenotype, and the development of tissue pathologies.

Thus, in our work, for the first time, a broad proteomic analysis of the matrisome of individual fractions of senescent MSCs, such as the ECM, CM, and cell lysate, was carried out. The matrisome analysis revealed significant changes in ECM production (Figure 7). A shift in production towards regulatory molecules and the significant downregulation of the main structural and adhesion molecules of the ECM, especially collagens, fibulins, and fibrilins, have been established. In addition, a decrease in the level of cathepsins, galectins, S100 proteins, etc. was found. It is worth noting that there was a significant increase in the regulatory molecules SERPINs, TIMPs, etc.

The observed alterations may be causally related to age-related pathologies. A number of downregulated core matrisome proteins are involved in the formation of focal contacts, transmission of the PI3K/AKT/mTOR signaling pathway, and binding of growth factors. Consequently, they may be associated with disturbances in adhesion, proliferation, growth, and migration in neighboring cells. MSCs are found in many tissues, with a particular abundance in the hematopoietic and perivascular niches. The modification of the MSC matrisome affects numerous populations of cells in the microenvironment, including endothelial cells, hematopoietic stem cells, and immune cells. A decrease in the representation of proteins with cytoprotective, anti-inflammatory, and antifibrotic properties was also observed. Conversely, the concentration of inflammatory proteins and regulators of profibrotic pathways has been observed to increase. Furthermore, the upregulation of proteins that can exert a direct prosenescent effect on microenvironmental cells (SERPINE1, THBS1, and GDF15) has been noted.

The secretome of senescent MSCs continues to be a subject of active investigation. Our study describes a number of ECM molecules that are associated with pathological changes during aging. However, the extent of their role for the implementation of prosenescent programs remains to be clarified in more targeted studies. Further research is required to elucidate the effects of identified senescence-associated ECM molecules on different cell types. This is essential for the modification of the aging niche and the development of protocols for regenerative medicine.

## 4. Materials and Methods

### 4.1. Cell Cultivation

In the study, ASC52telo was used. ASC52telo is an hTERT-immortalized adipose-derived mesenchymal stem cell line exhibiting a fibroblast-like morphology that was isolated in 2006 from the adipose tissue of a woman (ATCC^®^ SCRC-4000™). The cells were expanded in α-MEM (Gibco, Life Technologies, Carlsbad, CA, USA) with 50 U/mL penicillin-streptomycin (PanEco, Moscow, Russia), and 10% fetal bovine serum (FBS) (HyClone, Logan, UT, USA) at standard conditions (5% CO_2_, 37 °C). Subculture was carried out at 80–90% confluence of the cell layer. The cells were positive-stained with antibody against stromal markers CD90, CD73, and CD105. Accuri C6 flow cytometer was used (BD Biosciences, San Jose, CA, USA).

### 4.2. Cell Senescence Identification

Mitomycin C (MmC) was used to obtain stress-induced senescent MSC. A Petri dish was seeded with 250,000 cells. Cells (90–100% confluence) were incubated in complete growth medium containing 1.5 μg/mL MmC for 18 h, and then washed twice with PBS, and cultured for 10 days without reseeding. MSC without MmC served as the control.

For cell size and structure analysis, flow cytometric forward scatter (FSC) and side scatter (SSC) density plots were applied (CytoFLEX Flow Cytometer, Beckman Coulter Life Sciences, Indianapolis, IN, USA). The enlargement of cells and increase in the granularity are considered as specific features of cell senescence as well [99].

Autofluorescence data were acquired with CytoFLEX flow cytometer (Beckman Coulter Life Sciences, Indianapolis, IN, USA), using the excitation laser at 488 nm and detection optic at 525/50 nm [34].

Cell viability. The cells were trypsinised, and the suspension was stained with Annexin V–FITC kit (Immunotech, Marseille, France) according to manufacturer’s instructions. Cells were analyzed using Accuri C6 flow cytometer (BD, Franklin Lakes, NJ, USA).

Expression of senescence-associated β-galactosidase (SA-β-gal) activity at pH 6.0 was estimated with the Senescence Cells Histochemical Staining Kit (MilliporeSigma, Burlington, MA, USA) following the manufacturer’s protocol. We analyzed five view fields in each experimental point using an Eclipse TiU phase-contrast microscope (Nikon, Tokyo, Japan). Cell count was performed with Sigma ScanPro 5.0 Image Analysis Software (SPSS Inc., Chicago, IL, USA). Positive and negative cells were counted and the percentage of SA-β-gal+ cells was calculated.

To estimate proliferative potential, colony-forming unit assay was performed. The cells were seeded at low density (100 cells/25 cm^2^) and cultured for 7 days at 37 °C with 5% CO_2_. The cells were expanded in α-MEM (Gibco, Life Technologies, Carlsbad, CA, USA) with 50 U/mL penicillin-streptomycin (PanEco, Moscow, Russia), and 10% fetal bovine serum (FBS) (HyClone, Logan, UT, USA). After 7 days, the media was aspirated and adherent cells were washed with PBS; then, 1 mL ice-cold methanol was added to each well for 5 min to fix the cells. The methanol was aspirated and 2 mL Wright–Giemsa Stain (MilliporeSigma, Burlington, MA, USA) was added to each well and stained for 10 min. The stain was aspirated and the cells washed with PBS. The colonies were analyzed using a light microscope (Eclipse TiU phase-contrast microscope, Nikon, Tokyo, Japan).

### 4.3. Morphological Characteristics of “Senescent” ECM and Gene Expression Analysis

To analyze the production of extracellular matrix (ECM) by non-treated and senescent MSCs for 10 days, the following methods were used:

To detect collagenous and non-collagenous ECM proteins, 0.1% Sirius Red F3BA (Thermo Fisher Scientific, Waltham, MA, USA) or 0.1% Fast Green FCF (Thermo Fisher Scientific, Waltham, MA, USA) in saturated picric acid was used. The samples were incubated at room temperature for 30 min. Images were captured with Nikon Eclipse Ti-U microscope (Nikon, Tokyo, Japan). For semi-quantitative analysis, the dye bound to the ECM was dissolved with 200 μL of a mixture of 0.1% NaOH and methanol (1:1). The absorbance of solutions was determined as optical density on 550 and 620 nm wavelength using spectrophotometer (Bio-Rad Laboratories, Berkeley, CA, USA).

An enzyme-linked immunosorbent assay was used to assess the production of one of the most common types of collagen in connective tissue, collagen type I. Since collagen is an insoluble protein, we examined the content of its precursor pro-collagen 1α1 in the conditioned medium. For this purpose, we used the DuoSet ELISA kit (R&D Systems, Minneapolis, MN, USA) according to the manufacturer’s instructions.

The ECM components of MSCs were identified using immunocytochemistry. Cells were washed twice with PBS and fixed with 4% paraformaldehyde with 0.2% Triton X-100 (both Merck, Darmstadt, Germany) at 37 °C for 15 min. Nonspecific binding of antibodies was blocked with 1% bovine serum albumin (MilliporeSigma, Burlington, MA, USA) for 15 min at room temperature. Next, the cells were incubated with primary antibodies rabbit anti-human to collagen 1 (5 μg/mL; IMTEK, Moscow, Russia), fibronectin (20 μg/mL; IMTEK, Moscow, Russia), and human vitronectin (5 μg/mL Abcam, Cambridge, UK) overnight at +4 °C in a humid chamber. FITC-conjugated goat an-ti-rabbit secondary antibodies were used for detection (10 μg/mL; IMTEK, Russia). The preparations were mounted in Fluoroshield mounting medium containing Dapi nuclear dye (Sigma-Aldrich MilliporeSigma, Burlington, MA, USA). Samples were analyzed using a confocal laser scanning microscope LSM-900 (Carl Zeiss AG, Oberkochen, Germany).

The gene expression of structural components of the ECM: collagen type I (*COL1A1*), fibronectin (*FN1*), elastin (*ELN*), and ECM regulatory molecules: interstitial collagenase (*MMP1*) and type IV collagenase (*MMP2*), urokinase-type plasminogen activator (*PLAU*), and metalloproteinase inhibitor (*TIMP3*) were estimated. MSCs were lysed using the ExtraRNA reagent (Evrogen, Moscow, Russia). Using phenol-chloroform extraction, RNA was isolated and cDNA was synthesized on its template in a reverse transcription reaction. The concentration of mRNA and cDNA was measured using a NanoDrop spectrophotometer (Thermo Fisher Scientific, Waltham, MA, USA). The expression level of the above genes was assessed using real-time PCR using primers (Qiagen, Hilden, Germany). Changes in the mRNA level were assessed by the intensity of inclusion of the fluorescent dye SYBR Green (Synthol, Moscow, Russia). All gene expression levels were determined using the 2^−ΔΔCt^ method, and normalized to the *HPRT1* and *RPLP0* housekeeping genes.

### 4.4. Senescence-Related Changes in Proteome Composition of MSC Matrisome

#### 4.4.1. dcECM Sample Preparation

To analyze the extracellular matrix on cell-free preparations, the decellularization method was used. MSCs were washed thrice with PBS (PanEco, Moscow, Russia) and treated with 0.5% Triton-X100 + 20 mM NH4OH (both Merck, Darmstadt, Germany) in PBS at 37 °C for 5 min. Cell lysis was monitored using phase-contrast microscopy (Nikon Eclipse Ti-U, Tokyo, Japan). After incubation, cell debris was removed using PBS. To exclude DNA contamination, the preparations were treated with type I DNAse 50 U/mL (SciStore, Moscow, Russia) for 30 min at 37 °C.

Next, proteins soluble in 2 M urea in 150 mM NaCl solution (Thermo Fisher Scientific, Waltham, MA, USA) were extracted from decellularized ECM (dcECM) at 4 °C for 2 days with the addition of protease inhibitors, including metalloproteinases (Thermo Fisher Scientific, Waltham, MA, USA). The concentration of extracted proteins was measured using a bicinchoninic acid assay with bovine serum albumin as a standard (Pierce BCA Protein Assay Kit, Thermo Fisher Scientific, Waltham, MA, USA).

#### 4.4.2. Preparation of Conditioned Media Samples

The conditioned medium for the analysis was separated from the cells by centrifugation at 3000× *g* for 10 min, and then cleared from the cell debris by centrifugation at 16,000× *g* 4 °C for 10 min.

#### 4.4.3. Preparation of Cell Lysate Samples

The cells were washed from the medium in phosphate buffer twice; then, the cells were destroyed with cell lysis buffer (Lysis Buffer, Thermo Fisher Scientific, Waltham, MA, USA). To reduce the viscosity of the samples, 250 units of universal nuclease (Universal Nuclease, Thermo) were added and incubated at room temperature for 15 min. The samples were then incubated at 95 °C for 5 min.

#### 4.4.4. Preparation of Samples for Liquid Chromatography–Mass Spectrometric Analysis

The next steps in the preparation of all three types of samples (ECM, cell lysate, and conditioned medium) for mass spectrometric analysis were the same and consisted of reduction with 0.1 M dithiothreitol in 0.1 M Tris buffer (pH 8.5) containing 8 M urea, at 50 °C for 45 min, alkylation with 0.05 M iodoacetate, and incubation in the dark at room temperature for 20 min. The proteins were then precipitated overnight at −20 °C with five volumes of acetone in the presence of 0.1% trifluoroacetic acid. The protein precipitate was washed first with acetone, and then with 96% ethanol, separating the precipitate by centrifugation at 16,000× *g* at 4 °C for 10 min.

To the protein substrate sample, 100 μL of 0.05 M ammonium bicarbonate buffer and a solution of a mixture of trypsin and lysC with a concentration of 1 μg/μL in 0.1% acetic acid were added to a ratio of 1:100 in mass fractions to protein. The mixture was incubated overnight in a thermomixer at 37 °C with stirring at a speed of 750 rpm. Then, 1 μL of 10% formic acid solution was added to inactivate trypsin. Samples were centrifuged at 21,000× *g* for 10 min and an aliquot of the supernatant was transferred to a new tube for subsequent liquid chromatography–mass spectrometry analysis. The concentration of proteins in the samples within each group (ECM, lysate, and conditioned medium) was aligned.

#### 4.4.5. LC-MS/MS Proteomic Analysis

The resulting mixtures of tryptic peptides were analyzed by liquid chromatography–mass spectrometry based on a Dionex Ultimate3000 nano-HPLC system (Thermo Fisher Scientific, Waltham, MA, USA) and a TimsTOF Pro mass spectrometer (Bruker Corporation, Billerica, MA, USA). Peptides were separated using a packed emission column (C18, 25 cm × 75 μm × 1.6 μm) (Ion Optics, Parkville, Australia) at a flow rate of 400 nL/min by gradient elution from 4% to 90% of phase B during 40 min. Mobile phase A consisted of 0.1% formic acid in water, and mobile phase B consisted of 0.1% formic acid in acetonitrile.

Mass spectrometric analysis was performed using the Parallel Accumulation—Serial Fragmentation (PASEF) acquisition method [100]. An electrospray ionization (ESI) source was operated at 1500 V capillary voltage, 500 V end plate offset, and 3.0 L/min of dry gas at temperature of 180 °C. The measurements were carried out in the m/z range from 100 to 1700 Th. The ion mobility was in the range from 0.60 to 1.60 V s/cm^2^. The total cycle time was 1.88 s and the number of PASEF MS/MS scans was set to 10.

#### 4.4.6. Data Analysis

The resulting LC-MS/MS data were analyzed using PEAKS Studio 8.5. Each protein was identified by at least one unique peptide. For semi-quantitative analysis, the “label-free” method with the additional option “match between the runs” was used. The parameters were as follows: parent mass error tolerance—20 ppm; fragment mass error tolerance—0.03 Da; enzyme—trypsin; missed cleavages—3; fixed modifications—carbamidomethyl (C); and variable modifications—oxidation (M), and acetylation (N-term). False discovery rate (FDR) threshold was set to 0.01.

#### 4.4.7. Statistical and Bioinformatics Methods of Data Processing

Statistical analysis was carried out in the Statistica 12 program. Each group of samples (ECM, lysate, and conditioned medium) included both MmC+ and Mmc− samples. We normalized the data on the protein concentration in the samples within the group. The homogeneity of sample variances was assessed using the Levene’s test. The normality of data distribution in each group was determined using the Shapiro–Wilk test. In the absence of normal distribution and equal variances, the Mann–Whitney test, which is a nonparametric alternative to the *t*-test, was used.

Molecular functions and biological processes enriched were determined using DAVID (https://david.ncifcrf.gov, accessed on 15 February 2024) and STRING (https://string-db.org, accessed on 15 February 2024) web resources, and protein information was obtained from UniProt database (https://www.uniprot.org, accessed on 15 February 2024).

### 4.5. Statistical Analysis

A minimum of three independent experiments were performed for each assay. Analysis of group differences was performed by nonparametric Mann–Whitney test for independent samples using SPSS 14.0 software (SPSS Inc., Chicago, IL, USA). A level of *p* < 0.05 was accepted as statistically significant.

## Data Availability

Data are contained within the article.
